# Overview of Facial Plastic Surgery and Current Developments

**DOI:** 10.1055/s-0036-1572360

**Published:** 2016-02-04

**Authors:** Jessica Chuang, Christian Barnes, Brian J. F. Wong

**Affiliations:** 1 Beckman Laser Institute and Medical Clinic, University of California Irvine, Irvine, California; 2 Department of Otolaryngology–Head and Neck Surgery, University of California Irvine, Irvine, California

**Keywords:** facial plastic surgery, skin rejuvenation, rhinoplasty, craniomaxillofacial reconstruction, injectable fillers, facial reanimation, 3D imaging and printing, stem cells, wound healing

## Abstract

Facial plastic surgery is a multidisciplinary specialty largely driven by otolaryngology but includes oral maxillary surgery, dermatology, ophthalmology, and plastic surgery. It encompasses both reconstructive and cosmetic components. The scope of practice for facial plastic surgeons in the United States may include rhinoplasty, browlifts, blepharoplasty, facelifts, microvascular reconstruction of the head and neck, craniomaxillofacial trauma reconstruction, and correction of defects in the face after skin cancer resection. Facial plastic surgery also encompasses the use of injectable fillers, neural modulators (e.g., BOTOX Cosmetic, Allergan Pharmaceuticals, Westport, Ireland), lasers, and other devices aimed at rejuvenating skin. Facial plastic surgery is a constantly evolving field with continuing innovative advances in surgical techniques and cosmetic adjunctive technologies. This article aims to give an overview of the various procedures that encompass the field of facial plastic surgery and to highlight the recent advances and trends in procedures and surgical techniques.

The practice of modern facial plastic surgery began more than 100 years ago. 1 Otolaryngologists who believed in treating physical defects that caused patients psychological distress, social, and/or economic disadvantages pioneered the creation of facial plastic surgery as a subspecialty of otolaryngology. In the beginning, aesthetic surgery was outside mainstream medicine, but Jacques Joseph first promoted the value of cosmetic surgery as a specialized field. 1 2 Jacques Joseph is considered the founding father of modern facial plastic surgery, and he pioneered many of the earliest surgical aesthetic techniques that were later adopted and modified by fellow surgeons. 2 Another seminal contributor was Sir Harold Gillies, a New Zealand otolaryngologist by training who first standardized rhinoplasty, skin grafts, and facial reconstruction as described in his 1920 book *Plastic Surgery of the Face* . 3 He is often considered the founding father of plastic surgery. 3 4 5 The creation of the American Academy of Facial Plastic and Reconstructive Surgery (AAFPRS) in 1964 officially marked the beginning of modern facial plastic surgery as a subspecialty of otolaryngology. 6 Since then, facial plastic surgery societies have expanded globally to include the European Academy of Facial Plastic Surgery and the International Federation of Facial Plastic Societies, among others. 7

As a clinical specialty, facial plastic surgery is generally divided into cosmetic and reconstructive procedures, although many surgeons have broad practices encompassing both. In general, the scopes of practice for the majority of facial plastic surgeons in the United States are focused on cosmetic procedures (e.g., rhinoplasty, browlifts, blepharoplasty, facelifts) and reconstruction of defects in the face after skin cancer resection. Most facial plastic surgeons also use injectable fillers, neural modulators, lasers, and other devices aimed at rejuvenating skin. Facial plastic surgeons who focus on skull base and craniomaxillofacial trauma or microvascular reconstruction usually practice in tertiary centers such as university hospitals.

Facial plastic surgery is technically considered a subspecialty of otolaryngology head and neck surgery, and surgeons are diplomats of the AAFPRS. Facial plastic surgeons complete American Board of Medical Specialties–accredited residency training in otolaryngology as well as a 1- to 2-year facial plastic surgery fellowship. In contrast to general plastic surgeons, facial plastic surgeons focus on procedures and operations involving anatomy from the neck up. In reality, the specialty has significant overlap and crossover with general plastic surgery, oral maxillofacial surgery, ophthalmology, and dermatology.

Facial plastic surgery is a broad field of otolaryngology surgery that involves reconstructive and cosmetic techniques in addition to the use of biomaterials, lasers, and other adjunct materials to improve outcomes. This article is by no means comprehensive but gives an overview of the various procedures that encompass the field of facial plastic surgery, highlights recent advances and trends in interventions, and aims to identify the direction of future technologies and cosmetic agents.

## Cosmetic Surgery

Facial cosmetic surgery focuses on improving patient facial appearance. Common surgical procedures include rhinoplasty, blepharoplasty (eyelid surgery), rhytidectomy (facelift), browlift, genioplasty (chin augmentation), otoplasty (ear repositioning), liposuction, and fat transfer. Many patients seek surgical treatment to reverse changes that occur with aging, such as loose skin, decreased tissue volume around the face and neck, crow's-feet at the corner of eyes, fine lines on the forehead, loss of jawline contour, sagging jowl, and double chin.

### Rhinoplasty

Perhaps the most commonly performed and most difficult facial plastic surgery is rhinoplasty. It is performed to correct intrinsic and extrinsic nasal pathology, to modify unsatisfactory aesthetic appearance, to reduce airway obstruction (due to septal deviation, inferior turbinate hypertrophy, deviated/fractured nasal bones, and narrow internal nasal valve area), and to reconstruct congenital nasal anomalies. 8 During a rhinoplasty, the nasal skin, subcutaneous soft tissue, cartilaginous and bony framework, and mucous membrane lining are manipulated. 9 The open rhinoplasty is differentiated from the endonasal rhinoplasty in that the incision is made in the columella (fleshy tip of the nose that separates the nares) in the open approach. 9 The general principle of a rhinoplasty consists of a separation of the nasal skin and soft tissues from the osseocartilaginous nasal framework so that the framework can be reshaped to produce the desired nasal contours. Rhinoplasty is a technically challenging procedure that has complication rates from 4 to 18.8%. 8 Postoperative scarring and edema as well as patient dissatisfaction can lead to revision surgery (secondary rhinoplasty).

Over the past 10 years, rhinoplasty surgery has trended toward using structural techniques that require cartilage tissue to reconstruct shape, bolster anatomic components, expand the airway, and establish appropriate aesthetic contour. 10 Advances in this area include the broader use of cartilage for structural grafting. Traditionally, rib cartilage was used for only major reconstructive nasal operations, but more recently the number and type of grafts that are used in rhinoplasty have increased at least 10-fold as rib graft use became more prevalent in even primary cosmetic rhinoplasty. 11 Several new technological devices have evolved in recent years including polydioxanone foils to stabilize structural planes, 12 ultrasonic devices to perform precise osteotomies, 13 and the intranasal application of conventional high-speed powered instruments. 14 Digital imaging has become an increasingly important element of rhinoplasty planning, evolving to become an essential component of the preoperative consultation. Three-dimensional (3-D) imaging systems along with 3-D image morphing technologies are now widely used by most surgeons, though there is no universally accepted software platform. 15

### Facelift

The facelift is another common procedure performed by facial plastic surgeons. When facelifts were first advocated over 100 years ago, the procedure itself involved making multiple incisions and pulling the skin on the face tighter. Now the traditional incision is made in front of the ear, extending up into the hairline and curving around the bottom of the lobule and then behind the ear. 16 17 The skin is separated from the deeper tissues, and then the deeper tissues are tightened with sutures. In the final step, the skin is redraped with the excess skin removed. 16 18

Advancements in facelift procedures have been largely driven by the demands and desires of patients. Contemporary patients are looking for minimally invasive procedures with little to no postoperative downtime. Treatment of the aging face has been profoundly impacted by the rise of so-called lunchtime facelift-type operations. 19 20 21 In the mid-1990s, facelifts and rejuvenation operations were becoming increasingly technically demanding and risky due to exposure of the facial nerve branches and extensive soft tissue dissections. 22 These complex procedures yielded superb outcomes in expert hands; however, often patients operated on by less-experienced surgeons experienced prolonged postoperative edema, sensory or motor nerve injury, and facial asymmetry. 22 23 Today there are many facelift approaches, such as the deep plane facelift, composite facelift (which involves repositioning and fixation of the orbicularis oculi muscle), midfacelift, minifacelift, thread lift, periosteal facelift, skin-only facelift, and minimal access cranial suspension lift. 22 Each can achieve outstanding outcomes but largely depend upon surgical skill and the anatomic variations from patient to patient. In the 2000s, there was a rise in less invasive procedures, which could be performed under local anesthesia with oral benzodiazepines or with moderate sedation. 24 Overall numbers of facelifts increased because of the marketing of many nationally branded facelift-rejuvenation companies. According to the American Society for Aesthetic Surgery, the number of facelifts performed in 2014 grew by 27.7% compared with 1997. 25 A major trend in recent years is the combination of facelift operations with autologous fat transfer, which also addresses the volume loss that occurs with aging. 26

### Blepharoplasty

Another popular facial procedure is blepharoplasty, or eyelid surgery. Blepharoplasty involves the excision of excessive eyelid skin and/or removal of orbital fat to treat dermatochalasis (aging-related changes in the periorbital structures) and blepharochalasis (excessive papery thin skin). 27 The action of gravity on periorbital structures, decreased strength in periorbital muscles, sun damage, and changes in skin composition may cause aesthetically displeasing changes referred to in the vernacular as “droopy eyelids,” “tired eyes,” or “bags under the eyes.” The traditional approach for lower blepharoplasty is through a subciliary incision with a raised skin and muscle flap, followed by identification and correction of herniated medial, middle, and lateral fat. 27

The skin pinch blepharoplasty is the easiest to perform. In this technique, only excess skin is excised through a subciliary approach; it avoids a heavy skin–muscle flap, which can create worrisome vertical traction and swelling in the periorbital tissues. 28 The pinch blepharoplasty of the lower lid also foregoes violation of the orbicularis muscle and the orbital septum to avoid nerve injury and to decrease scarring. 28 This approach allows more wrinkled, thin skin to be safely removed and an aesthetic eyelid posture to be maintained. Both upper and lower lid blepharoplasties are often performed under local anesthesia or moderate sedation. Of note, blepharoplasty is also performed to correct lid ptosis, which results from several causes. 29 The most common cause is levator palpebrae superioris attenuation. 30

### Forehead Lifts and Browlifts

Forehead lifts or browlifts also are a large part of a facial plastic surgery practice. This procedure is relatively straightforward in terms of its classical approach where excess skin is resected and the forehead skin is repositioned superiorly. 31 The incisions are placed along a coronal line within the hair-bearing scalp if the frontal hairline is low. A hairline (trichial or trichophytic) incision is used in patients with a high hairline. 32 These two approaches are most commonly used in women. In men, a third option is the midbrowlift where an incision is made in a deep brow furrow, and a fusiform ellipse of skin is resected. 32 A direct browlift, where the incision is made at the upper margin of the eyebrows, is rarely performed. Both mid- and direct browlifts may leave a visible scar, and they are more commonly used for functional brow surgery in patients who have significant brow ptosis contributing to a mechanical visual field defect. The newest approach is endoscopic surgery where several small incisions are placed behind the hairline and an endoscope is used for visualization during elevation of the forehead skin. With the endoscope under the forehead flap, the surgeon releases soft tissue from the arcus marginalis and temporal line of fusion, allowing redraping and fixation of the skin more cephalically. 33 34 The results are less dramatic, but it is an outstanding approach in the younger patient who seeks more natural and less-defined changes.

### Hair Restoration

Hair loss is a major concern for many, and hair transplantation advancements continue to provide a good option for correction. 35 Numerous physicians across a dozen specialties perform these procedures. Historically, punch grafts of the scalp were used in hair transplantation, but these created a highly unnatural look over the recipient area with multiple scars at the donor site. The concept of minigrafts and micrografts was advocated in the late 1970s. 36 Scalp flap surgery was relatively popular during the 1980s, but in 1984 Headington pioneered the concept of follicular unit transplantation. 37 Follicular unit transplantation is now the gold standard of hair restoration. It involves individual hair follicles or small groups of two to four follicles to be extracted and transplanted. It creates the most natural outcomes. 35

Robotic surgery has definitely made advances across multiple surgery specialties and is now being used to automate follicular unit harvest and transplantation. 38 39 Semiautonomous robotic systems harvest, sort, and process individual single hair grafts or multiple hair grafts. 40 It remains to be seen whether these systems are economically viable related to conventional methods, which required meticulous sectioning of follicular units by teams of technicians.

## Nonsurgical Cosmetic Procedures

A large evolving part of facial plastic surgery involves using techniques such as chemical peels, lasers, and various injectable substances to produce improved facial aesthetics. In comparison with most cosmetic surgery, these office-based procedures have a much-reduced recovery period.

### Chemical Peels

Examples of chemical peel agents include glycol acid, trichloroacetic acid, and phenol (listed from mildest to strongest). 41 42 During a chemical peel, the agent penetrates through the epidermis into the first layer of the dermis. Different agents have different depths of penetration and are divided into four histologic grades. 43 The chemical agent causes destruction at various depths of the skin and stimulates skin regenerative pathways in the dermis that are not well understood. 44

### Dermabrasion

Dermabrasion is another technique used to smooth deeper scars and wrinkles. This technique is performed under a local anesthetic and/or a freezing agent. A high-speed rotating brush, sandpaper, or similar abrasive device is used to remove the top layer of the skin. 45 This technique may be applied to individual blemishes or large areas of the face.

### Lasers

Lasers are used to correct facial rhytides, scarring, photo-damaged skin, and other signs of aging. Laser treatments cause little to no bleeding and induce minimal trauma to the surrounding skin. The technology allows for precise control of ablation depth. There are two types of laser therapy: ablative and nonablative.

Ablative lasers vaporize the superficial layers of the skin by heating the dermis to stimulate new collagen production by fibroblasts. Of the ablative lasers, there are two laser wavelengths in common use: pulsed carbon dioxide (CO _2_ ) and erbium:yttrium-aluminum-garnet (Er:YAG). On average CO _2_ systems ablate 20 to 60 μm of tissue with the initial pass, and the residual thermal damage extends to a depth of 20 to 100 μm after multiple passes. 46 The Er:YAG pulse laser systems vaporize 2 to 5 μm of tissue per pass and leave behind a 20- to 50-μm zone of residual thermal damage. 46 Thus erbium laser systems can be more finely tuned than CO _2_ systems and are useful for precise, finely adjusted, light to medium ablations. CO _2_ systems are more efficient for deep ablations.

Currently, novel systems are being developed to deliver simultaneous irradiation from combined Er:YAG laser and CO _2_ laser, variable-pulse Er:YAG, and dual ablation/coagulation Er:YAG. 47 48 These new systems aim to achieve a significant degree of clinical improvement with less skin wounding to produce granulation tissue and fibroplasia but with thin zones of thermal damage. 47 Some complications of ablative lasers include pain, edema, persistent erythema, infections, postinflammatory hyperpigmentation, and hypopigmentation. 46

Nonablative lasers do not cause superficial injury to the epidermis but instead only stimulate collagen growth by creating focal thermal injury within the dermis. 49 50 The Q-switched neodymium:yttrium aluminum garnet laser is especially useful in the treatment of periocular and perioral rhytides. Some other nonablative lasers include diode lasers, pulsed-dye lasers, and intense pulsed nonlaser light sources. 49 51 Much research remains to be performed to better understand the laser–tissue interaction caused by nonablative lasers.

Finally, one of the biggest advances within the past 10 years is the development of spatially selective heating of tissue or fractional photothermolysis. The concept consists of thermal injury in full-thickness columns of controlled depth and width (microthermal zones), leaving the areas of untreated skin between them, which enables rapid tissue proliferation to repair the damaged areas. 52 Fractional photothermolysis causes deep dermal damage that triggers collagen synthesis and remodeling while causing minimal epidermal damage. 49 There are both fractional nonablative lasers and fractional ablative lasers. In general, the obtained therapeutic results of fractional nonablative lasers are better than those of nonablative lasers but are still inferior to traditional ablative resurfacing.

Fractional ablative lasers combine the principles of classic ablative techniques and fractional photothermolysis. The laser beam of ablative fractional lasers causes microscopic treatment zones consisting of central tiny ablative foci (microscopic ablation zones), surrounded by a thin necrotic zone encased by a coagulation zone (microscopic coagulation zone). 49 53 54 55 Between the microscopic treatment zones, are areas of untreated skin ensure rapid healing. Re-epithelialization occurs within 48 hours with extrusion of the necrotic tissue with minimal inflammatory reaction. The postoperative erythema usually resolves in 7 days. 49 The main advantage of fractional ablative resurfacing is its low risk for side effects and complications. Fractional laser therapy was a great revolution in the field of laser skin resurfacing, and more laser devices are currently being developed and commercialized.

### Evolving Paradigm

One of the most important changes in facial rejuvenation has been a shift from a two-dimensional focus on hyperdynamic facial lines and the immobilization of corresponding muscles to an increased understanding and appreciation of the three-dimensional aspects of facial aging, in particular the loss of volume. 56 Facial rejuvenation now encompasses areas of movement control, recontouring, and volume restoration using multiple modalities. In conjunction with the recent trend toward minimally invasive cosmetic improvements in facial rejuvenation, clinicians are now using combinations of neuromodulators (distinct serotypes outlined below) with dermal fillers such as hyaluronic acid to address facial rejuvenation and to increase the longevity of the outcomes. 57 Botulinum toxin remains a popular neurotoxin used to paralyze muscles to treat wrinkles caused by muscle activity. Injectable fillers are used to replace lost volume in the face and are available in increasing varieties. The most widely used filler products are autologous fat, collagens, hyaluronic acid, and synthetic polymers. 56 58

### Fat Grafting

Complementary fat grafting took hold broadly across the specialty as years of evidence largely driven by the work of many surgeons and dermatologists demonstrated the efficacy of the manual liposuction of fat and precise injection of small aliquots of this tissue into the regions surrounding the midface, orbit, temporal fossa, neck and other regions. Fat grafting is an essential component of many individuals' facelifts now, and there is much discussion and debate about whether mesenchymal stem cells are transferred in this process as well. 59 Fat grafting can be performed using local anesthetic or moderate sedation. One issue with autologous fat grafting is the variability of graft survival (10 to 80%), but there is continued research in improving fat transplant survival. 60 Some techniques include pharmacologic manipulation of grafts with insulin-like growth factor, basic fibroblast growth factor, selective β-1 blocker, and platelet-rich fibrin matrix to enhance the survival of lipografts. 61 62 The regulatory environment concerning these modifications is quite complex.

### Dermal Fillers

Replacing lost or misplaced fat pads with dermal fillers is one important approach to recontouring the aging face as well as to treating scars and rhytides. The U.S. Food and Drug Administration (FDA)-approved temporary fillers consist of collagen, hyaluronic acid, calcium hydroxylapatite (CaHA), and poly-L-lactic acid (PLLA). 58 The only FDA-approved permanent filler is polymethyl methacrylate for correcting nasolabial folds. PLLA and CaHA are synthetic fillers, laboratory-made substances that are not related to substances found in the native skin.

### Collagen

Collagen is a major component of human connective tissues, such as skin, cartilage, vasculature, and bone. The injectable forms of collagen can be purified from either bovine or human sources. These products are seldom used and were voluntary withdrawn from the U.S. market in 2010. They have since been supplanted by hyaluronic acids. 58

### Hyaluronic Acid

Currently, hyaluronic acids are the dominant facial filler agent. Hyaluronic acid is also a major component of connective tissues, especially in the human dermis. It hydrates, lubricates, and stabilizes connective tissues, and as skin ages, the amount of hyaluronic acid decreases in tissues. Hyaluronic acid contains no products of animal origin, and therefore eliminates the need for preinjection skin testing. 63 The available hyaluronic acid products differ in their concentration, cross-linkage, and viscosity. Recent research focuses on the methods to produce more stable forms of hyaluronic acid that have longer in vivo retention times. 64 The main patient concerns about dermal filler injections are pain and discomfort as well as postprocedural bruising and swelling. There is an increasing trend toward adding lidocaine solution to fillers and using microcannulas for injection techniques. 58

### Poly-L-Lactic Acid

PLLA is a deep tissue regenerator that provides soft tissue augmentation through stimulation of fibroblast production. It is a synthetic polymer that stimulates collagen synthesis through a foreign-body reaction. It was the first dermal filler developed to treat facial lipoatrophy in patients with human immunodeficiency virus. In general, PLLA is frequently and successfully used to treat hollowing of the cheek, nasolabial folds, prejowl folds, the malar area, and the temporal area. Studies have indicated that results last at least 3 years with additional treatment sessions, and patient satisfaction is high. 65

### Calcium Hydroxylapatite

CaHA is another synthetic, nonanimal, inorganic compound that is not immunogenic. In 2006, CaHA was approved by the FDA to treat lipoatrophy via subdermal implantation to correct moderate to severe facial wrinkles and folds. Corrective results last up to 12 to 20 months after injection. 66

### Polymethyl Methacrylate

The only FDA-approved permanent filler is polymethyl methacrylate. 58 The main concerns of permanent fillers are the possibility of late-onset adverse events or displacement of the filler as facial structures change with age. Bellafill (Suneva Medical, Inc., Santa Barbara, California) is a third-generation filler that is composed of a suspension of polymethyl methacrylate beads in a bovine collagen delivery system with 0.3% lidocaine. The beads are not absorbed by the body but cause fibrotic granulation under the skin through foreign-body reactions. The beads are eventually encapsulated by endogenous collagen.

### Botulinum Toxin

Botulinum toxin injection for treatment of facial wrinkles is the most frequently performed cosmetic procedure in the United States. 67 The *Clostridium botulinum* bacterium produces eight serotypes of botulinum toxin protein (A, B, Cα, Cβ, D, E, F, and G). The most potent is the botulinum toxin serotype A, which is the one used for cosmetic treatments of glabellar lines and other hyperfunctional facial rhytides. 68 Botulinum toxins inhibit the release of acetylcholine into the synaptic cleft and therefore result in temporary muscle paralysis. The clinical effects generally last ∼2 to 6 months. 69 There are three botulinum toxin A (BoNTA) serotypes approved by the FDA for cosmetic use: onabotulinumtoxinA (BOTOX Cosmetic, Allergan Pharmaceuticals, Westport, Ireland), abobotulinumtoxinA (Dysport, Ipsen Biopharm Ltd., Wrexham, United Kingdom), and incobotulinumtoxinA (Xeomin, Merz North America, Raleigh, North Carolina). 70 There are five sources of botulinum toxin available worldwide. 71 These various preparations of botulinum toxin are neither identical nor interchangeable as their reconstitution in various solutions, storage temperature, and properties after dilution differ. BOTOX is 4 times more potent on a per unit basis than Dysport, and both are used for cosmetic purposes. Xeomin is a highly purified formulation that is free from complexing proteins, reducing its immunogenic potential. 72 In addition to treating already developed wrinkles, BoNTA has been shown to prevent the development of wrinkles. 69

## Reconstructive Surgery

Facial reconstructive surgery aims to correct anatomic defects and may include scar revision, craniomaxillofacial fracture repair, laceration repair, vascular malformation treatment, craniofacial and maxillofacial cleft operations, orthognathic surgery, and cancer reconstruction. Examples of cancer reconstruction include free flaps for head and neck cancer defects and local skin flaps for cutaneous tumors.

### Facial Fractures

Facial fractures are often due to trauma and can be divided into three types of injuries: Le Fort fractures, zygomaticomaxillary complex (ZMC) fractures, and mandibular fractures.

Le Fort facial fractures of the midface are complex and classified into three categories. They all involve fracture of the pterygoid plates. Le Fort I fractures (horizontal) often result from a directed force on the maxillary alveolar rim in a downward direction. 73 There is no orbital involvement in type I fractures. Le Fort II fractures (pyramidal) result from a blow to the lower or midmaxilla and are defined in part by separation of the maxilla from the cranial base at the zygomatic arch. Le Fort III fractures (transverse), also termed *craniofacial disjunction* , are often caused by an impact to the nasal bridge and are the most complex and devastating of the Le Fort fractures. These fractures are accompanied by severe intracranial trauma and result in complete separation of the facial skeleton from the skull base.

ZMC fractures are the second most common facial fractures after nasal fractures and are also referred to as *malar* or *cheekbone fractures* . The most common causes of ZMC fractures include assault, falls, motor vehicle accidents, and sports injuries. The ZMC provides normal cheek contour and separates the orbital contents from the temporal fossa and maxillary sinus. ZMC fractures are classified into type A1 (zygomatic arch), type A2 (lateral orbital wall), type A3 (inferior orbital rim), type B (involving all four anatomical sites), and type C (complex fractures with comminution of the zygomatic bone). 74

Mandibular fractures are frequent injuries after facial trauma due to the mandible's angularity and relative lack of structural support. Mandibular fracture types can be classified by injury of the anatomic regions: condyle, coronoid process, ramus, angle, body, alveolus, parasymphysis, and symphysis.

### Management of Facial Fractures

The ultimate goal in treating facial fractures is to obtain an accurate and stable reduction while minimizing external scars and deformity. For ZMC injuries that have fracture instability or comminution, an open reduction and internal fixation is indicated with the coronal approach as the main access for complex zygomatic arch repairs. This approach can lead to alopecia, loss of sensation posterior to the incision, risks of traction injury to the frontal branch of the facial nerve with temporal hollowing, and excessive blood loss. More recently, endoscopy has been used to assist in the treatment of ZMC and Le Fort III fractures. Endoscopy can be performed through stab incisions and thus avoids the need for extensive coronal exposure for zygomatic arch repairs. 75 Endoscopy allows for in situ reduction and fixation under magnified visualization while only requiring small, well-concealed incisions.

Mandibular fractures were traditionally treated with closed reduction or open reduction with wire osteosynthesis. In addition, rigid internal fixation was developed and involved placement of interfragmentary titanium plates.

The main advances in facial reconstruction for these traumatic facial fractures are in new instruments and hardware. Individual anatomy is recreated on a computer using computed tomography data. Customized plates are designed, and the operative plan is simulated on a computer workstation. This technology allows surgeons to better plan the operation, showcase expectations to patients, and teach fellow surgeons. Patient-specific implants in various materials including titanium and silicone have now been developed and utilized. 76 Additionally, resorbable plates and screws have been used in the treatment of mandibular fractures. 77 78 79 These plates are of particular use in children, whose facial bones continue to grow. Rigid fixation techniques now more commonly utilize smaller fixation plates instead of larger plates. 80 81

### Facial Reanimation

Facial paralysis can be a consequence of traumatic facial nerve injury, iatrogenic injury, oncologic resection, temporal bone surgery, skull-base surgery, congenital syndromes, and viral infections. 82 Facial reanimation surgery involves using surgical techniques to improve the facial deformity caused by facial paralysis with the goal of improving facial symmetry or restoring mimetic function. There are five types of repair: (1) neural techniques; (2) musculofascial transposition techniques; (3) microneurovascular transfer; (4) facial plastic surgery procedures; and (5) use of prosthetics. 82 83 Dynamic procedures aim to restore voluntary movement. These include cranial nerve XII–to–cranial nerve VII nerve transfers, interpositional grafts, cross-facial nerve grafts, dynamic musculofascial transpositions, and free flaps with cross-facial nerve grafting for reconstruction. Static procedures to treat facial paralysis include the use of upper eyelid alloplast implants, fascia lata slings, facelifts, browlifts, and eyelid procedures. 82 These techniques can provide marked cosmetic improvement and can restore function, particularly in eye protection, mastication, and speech.

### Microtia

Microtia is a congenital malformation of the external ear, which results in a disorganized remnant of cartilage in the auricle. Reconstruction may be performed through a multistage process consisting of the implantation of a rigid framework and the subsequent creation of the ear lobe and crease behind the ear. Reconstruction of the external ear can be performed with prosthetic ear replacement, prosthetic frameworks, and autologous cartilage. Historically, microtia repair was a four-stage operation with (1) procurement of cartilage of the chest wall; (2) construction and placement of cartilage framework; (3) lobule rotation, conchal excavation, and tragus formation; and (4) elevation of the pinna. 84 With recent advances and refinements in framework carving and techniques, it is a two-stage reconstruction process today: (1) creation of a 3-D costal cartilage framework and (2) ear elevation operation. 85 One study that compared the use of rib cartilage versus porous polyethylene implant for microtia reconstruction showed neither material to be superior; however, the polyethylene implants achieved a better cosmetic outcome in terms of ear definition, shape, and size. 86 The downside was that polyethylene implants had a higher risk for infection and extrusion. 86 Research in tissue engineering is currently being performed, and newer alloplasts are being used with aspirations that the use of engineered tissues and alloplasts may one day replace autologous cartilage.

### Otoplasty

Otoplasty is the surgical procedure to treat congenitally prominent ears. Otoplasty can be either cartilage splitting or cartilage sparing. Cartilage-splitting techniques involve incisions through the cartilage and repositioning of large blocks of auricular cartilage. Cartilage-sparing techniques avoid full-thickness incisions but create angles and curls in the cartilage for contouring. Most surgeons now perform cartilage-sparing otoplasty. 87 88

## Future Direction of Facial Plastic Surgery

Current trends in facial plastic surgery include increased utilization of nonsurgical techniques such as fillers and neurotoxins to treat the aging face, development of new laser technologies, utilization of 3-D imaging techniques for individualized plating in maxillofacial surgery in trauma, and minimally invasive techniques such as endoscopic approaches to minimize scaring.

### Combination of Fillers and BOTOX

BoNTA has now been a popular agent for cosmetic procedures for over 20 years. Its injection has proved to be safe, with a high degree of satisfaction. BoNTA research studies its effects and safety for expanding its indications for medical treatments. 89

In addition, there is research in development of various combination therapies with BoNTA and other modalities including fillers, intense pulsed light, laser modalities, and dermabrasion. 90 91 92 93 Aging is a complicated multifactorial process that causes undesirable wrinkles, furrows, sagging, dyspigmentation, and changes in skin textures. Two major causes of the aging processes are volume loss and muscular hyperactivity. There is current research and development in combination treatments with dermal fillers and BoNTA to simultaneously restore volume and relax muscle pull to more effectively recontour the face. 94 Combining BoNTA with intradermal fillers such as hyaluronan not only gives an immediate resting result but also allows the effects of the filler to last twice as long. 95 96

Moreover, Revance Therapeutics, Inc. (Newark, California) is developing a new BoNTA product called RT002, a novel injectable formulation of BoNTA that consists of a purified neurotoxin and a patented TransMTS peptide. 97 This product is designed to remain in the targeted treatment area and limit the spread of BoNTA to adjacent muscles. This new peptide is hypothesized to allow higher targeted doses of BoNTA. Based on the preclinical data, the RT002 durability is up to 7 months, compared with the average 2 to 6 months of current BoNTA sources. 97

Injection techniques and patterns in BoNTA applications are also evolving. 98 An increasing number of procedures are performed on the middle and lower face rather than just on the upper face. 96 The combination of BoNTA and hyaluronic acid filler is now the standard approach for the lower face. Another minimally invasive facial rejuvenation technique has been the use of BoNTA with augmentation and facial sculpting using autologous adipose tissue, which has been shown to extend results. 96 Blunt cannulas are now used by many providers to distribute fillers after an initial puncture is made through the skin allowing injection in multiple vectors with reduced needle trauma.

### Three-Dimensional Imaging

One of the most exciting trends in facial plastic surgery is the development of computer-guided 3-D reconstruction techniques. For example, computer-guided orbital reconstruction with mirror image overlay navigation improved outcomes in complex orbital reconstructions. 99 In addition, surgical navigation technology has been shown to be a new effective auxiliary method for improving the treatment results for zygomatic fracture.

### Three-Dimensional Printing

3-D printing technology is an expanding and innovative field in medicine and surgery. 100 3-D printing-based tactile prototype models are helpful in skull reconstruction, correction of craniosynostosis, simulating Le Fort osteotomies, and creation of ideal orbital wall meshes for orbital wall repairs. Facial reconstruction is a complicated procedure that often requires significant intraoperative time in contouring the titanium plates used to link adjacent bone together while the patient is under anesthesia. By creating a 3-D replica of the individual patient's bony anatomy, these titanium plates can be contoured before surgery and reduce operation time. 101 102 In addition, this method allows the creation of a precise fit, resulting in an improved aesthetic form, and reduces the risk for corrective surgery. 102 Bedside 3-D printing is another emerging technology and can create 3-D prostheses and models for individualized patient education, allowing preoperative surgical planning, creating patient-specific intraoperative guidance tools, and fashioning patient-specific implants. 103

### Endoscopy

As mentioned previously, there is a popular trend in facial plastic surgery toward minimally invasive techniques. Endoscopy has been shown to be safe and effective in the management of orbital floor fractures and zygomatic fractures. 104 105 Endoscopy allows good visualization of the anatomy without the need for extensive access incisions. 106 It minimizes scalp scar, decreases forehead numbness, shortens hospital stay, and provides a faster, more comfortable postoperative recovery for the patient. 105

### Advances in Biomaterials and Bioengineered Interfaces

The use of rigid implants is crucial for adequate bone immobilization in skeletal facial reconstruction. Historically, there have been postoperative complications of hardware use including pain from plates, loosening of screws, palpability of metal plates, temperature sensitization due to implants, increased maxillary and frontal sinus infections, radiographic artifacts, and hypersensitivity reaction to various alloys. 78 The use of nonmetallic materials would reduce these postoperative concerns, and research in this area is promising. Resorbable fixation material has been well studied in pediatric craniofacial surgery with good results. 79 107 Eppley studied the use of plates and screws composed of a specific poly L-lactic acid-polyglycolic acid material, concluding it can be effectively and safely used in the midface. 78

### Tissue Engineering

Repair of craniofacial injuries due to trauma, tumor removal, or congenital abnormalities is a large aspect of facial plastic surgery, and there is a need for both functional and aesthetic restoration of a complex variety. 108 Autologous grafts such as those harvested from the cranium, fibula, or iliac crest are the gold standard for head and neck reconstructions. However, autologous graft harvests are limited in availability, cause donor site morbidity, lack precision in contouring of donor graft shape, and have an inherent difference in the structure and biomechanics of the donor site and graft site. Engineering of personalized human bone grafts is an exciting field of facial plastic surgery. The key strategies involve the use of bioactive scaffolds, cell-seeded scaffolds, and autologous bone grafts grown in vitro. 108 Bioactive acellular scaffolds utilize biodegradable synthetic materials with osteoinductive factors that induce the recruitment of host cells into the scaffold and guide host cell–bone ingrowth. 108 Cell-seeded scaffolds combine exogenous cells usually harvested from bone marrow with bioactive molecules in scaffolds. These types of scaffolds seeded with bone marrow cells and platelet-rich plasma were shown to enhance osteogenesis. 109 110 One famous example of successful tissue engineering is the Vacanti total external ear reconstruction with a polyglycolic acid polylactic acid construct with seeded chondrocytes. 111

### Advances in Facial Reanimation

Current management of facial reanimation uses static biomaterial implants such as polymer or allograft tissue matrices to support de-enervated sagging tissue or microsurgical transfers of muscle to give movement to the paralyzed side. There is research into regenerative implants that could be composed of autologous myocytes cultured on matrix scaffolding that can respond to neural input for control. 112 There is also research in tissue-engineered constructs that have the potential to regenerate cells in various scaffolds as well as development of novel neuromuscular interfaces and replacement tissues for restoring facial function to create a more natural result in facial reanimation surgery. 113 Further studies are exploring neuroelectrical prosthetic devices to drive paralyzed musculature based on cues from the healthy side. For example, Ledgerwood et al developed an electroactive polymer artificial muscle device that could potentially restore lost muscular contraction for use as an eyeblink prosthesis. 114

In terms of advancements in surgical techniques, there are procedures being developed to drive the native facial muscles with the masseteric branch of the trigeminal nerve with minimal injuries to the donor site, as well as studies on using a cross-facial nerve grafts for the reanimation of the paralyzed face. 115 116 117 118 Cross-facial nerve grafts utilize the contralateral healthy facial nerve to reinnervate the paralyzed muscles. 119 120

There is also extensive basic science research in improving the management of facial reanimation that focuses on improving axonal transversement of nerve grafts, decreasing detrimental neural oversprouting and hyperstimulation, and improving the target accuracy of the regenerated nerve. 121 The “facegram” is a new tool that is being developed to acquire 3-D videographs of patients performing a set of facial expressions and converting the data into graphical information for analysis. Along this line of research is the development of a central database warehouse to allow physicians to upload individual patient facegram data. In the future, the central data set could help physicians better determine the optimal approaches for individual patients because it would offer a reference of available approaches and postoperative results that would augment individual practice experiences. 121

### Facial Reconstruction and Transplantation

Severe trauma to the face after burns or bullet trauma can be disfiguring and difficult to treat because the face is a complex entity in its form and function. 122 Over time, there have been advancements from using local soft tissue flaps and rigid fixation in reconstruction to the invention of microsurgical free tissue transfer, which allows clinicians to repair complex facial defects using free flaps and replace significant amounts of missing tissue. 122 The advantage of free flaps is that they can be modified extensively for better reconstructions. Most of the face and neck are composed of fasciocutaneous structure and can be more easily repaired via microsurgical procedures; however, injuries involving neuromusculature of the midface may warrant a facial transplantation. 122

The midface is a critical component of the face; it is the central region where facial movement is initiated. This central neuromuscular component preserves the individual's identity, and movement in this area must be natural postreconstruction. 122 Facial transplantations are more technically challenging than free flap transfers. The essential concept in facial transplantation is that of angioneurosome, which is the idea that the design of the flap should be based on blood supply, neuromuscular unit, and muscular origin and insertion, as well as preservation of rigid facial structures and cutaneous ligaments. 122 Facial transplantation allows transferring of various skin, soft tissue, and bone that can replace the lost tissue of the patient with an exact anatomic and functional match, but there are risks associated with immunosuppressant use, which increase the risk for infections, malignancies, and end-organ toxicities. 122

The first near-total face transplantation in the United States was performed at the Cleveland Clinic in 2008. Since then, several partial and total face transplantations have been performed in the United States. After a face transplantation, the patient may look similar to the donor but because of variations in skeletal structure and facial shape of the recipient, the result is a hybrid of the two faces. 122

### Stem Cells

Regenerative medicine plays an important role in many aspects of medicine and surgery. In the field of facial plastic surgery, stem cells are often used for bony and soft tissue defects, nonhealing wounds, and skin rejuvenation. 123 One example is adipose tissue–derived stem cells that secrete angiogenic growth factors like vascular endothelial growth factor, which have been shown to aid in marked improvements in survival rate of transplanted fat grafts. 124 Stem cells have also been shown to accelerate wound healing, reduce scar appearance, and prevent allotransplant rejection. 125 Adipose tissue–derived stem cells can be isolated from lipoaspirate blood and saline fraction in as short as 30 minutes. 126 Unfortunately, its quick availability and popularity have led to increased marketing of false “stem cell therapies” and scams. 127

### Wound Healing

Last but not least, wound healing is an important aspect of facial plastic surgery. It is crucial to maintain the basic principles of wound care, especially in the face. The principles include minimizing infection, maximizing tissue oxygenation, ensuring adequate nutrition, and determining adequate debridement when indicated. However, over the last decade, there have been advancements in adjunctive products to aid in wound healing. Multiple biological, synthetic, and genetically derived factors; bioengineered tissue substitutes; vacuum-assisted closure devices; and hyperbaric oxygen therapies allow clinicians to proactively treat facial wounds and reduce wound-healing complications, such as infection and scar formation. 128

Growth factors are one adjunctive product to enhance healing of wounds. Examples of growth factors include recombinant human platelet-derived growth factor, anti-deltalike ligand 4, and recombinant human transforming growth factor β-3. 129 Recombinant human platelet-derived growth factor has been used by otolaryngologists to improve wound healing in patients who have undergone irradiation. The irradiation of wounds may alter growth factor presence and create higher levels of growth factor inhibitor. 128 Anti-deltalike ligand 4 is a growth factor that increases tissue vascularity and thereby increases the rate of wound healing. 130 Intradermal injection of recombinant human transforming growth factor β-3 has been shown to reduce scarring. 128

Biologic products such as platelet-rich plasma, platelet-rich fibrin matrix, and autologous platelet-rich gel are also used to enhance healing. Platelet-rich plasma reduced infection in acute wounds and enhanced wound healing in chronic wounds. 131 Some studies suggested that platelet-rich fibrin matrix injection of the dermis may activate fibroblasts, induce collagen deposition, and increase angiogenesis of the wound. 132 Autologous platelet-rich gel has also been shown to significantly increase wound closure rate. 128

Other adjunctive techniques to enhance wound healing that are still in early research phases include vacuum-assisted closure, bioengineered skin, shock-wave therapy, pulsed radiofrequency wound healing, hyperbaric oxygen, and fetal wound healing. Of these, vacuum devices and hyperbaric oxygen therapy are the most studied. Vacuum devices improve blood flow by increasing the pressure gradient between the interstitial space and newly forming capillaries. It also removes excess fluid, reduces edema, decreases bacterial load, and increases granulation in the tissue bed. 128 Hyperbaric oxygen stimulates angiogenesis and fibroblast proliferation by causing vasoconstriction and increased partial pressures of oxygen in the blood. 128

As for bioengineered skin for facial plastic surgery, research in this field is limited. Currently there is one product called Apligraf (Organogenesis, Inc., Canton, Massachusetts) available as a permanent composite of dermoepidermal graft that is cultured from neonatal forehead skin, which has shown potential in enhancing wound healing. 128 Shock-wave therapy has been shown to induce expression of several genes and increase the production of growth factors that promote wound healing. 133 Studies in mice showed that pulsed radiofrequency treatment increases wound contracture and tissue granulation. 134 Perhaps the most novel concept in wound healing is that of fetal wound healing. In fetal wounds, there is more type III collagen instead of type I, hyaluronic acid is expressed in higher amounts, modulators of collagen creation are upregulated, and inflammatory mediators are decreased in fetal tissue. 135 136 Due to these differences, fetal skin repairs itself quickly and without scarring. Although scarless healing is still far in the future, the application of these concepts in fetal wound healing to adult wound healing may one day produce scarless healing in adults. 128

## Conclusion

Facial plastic surgery is a broad field of otolaryngology surgery that involves reconstructive and cosmetic surgery techniques in addition to biomaterials, lasers, and other adjunct materials to improve surgical outcomes. We hope that this article has provided a good overview of facial plastic surgery and previewed the upcoming advances in the field of facial plastic surgery. There are exciting research studies in the fields of dermal fillers, 3-D imaging and printing, endoscopy, new biomaterials and tissue engineering, new surgical techniques in facial reanimation and facial transplantation, stem cell research, and enhancements in wound healing. In conclusion, facial plastic surgery is an important field of surgery that can make lifesaving and life-changing transformations in patients' individual lives and in society. It is an ever-evolving field that leads innovation in surgical techniques, technological and computer-based advancements, biomaterials research, and minimally invasive nonsurgical and surgical procedures for facial rejuvenation and reconstruction.

## References

[1] Bhattacharya S Jacques Joseph: father of modern aesthetic surgery Indian J Plast Surg 2008 41(Suppl):S3 S8 20174541 PMC2825133

[2] Behrbohm H Briedigkeit W Kaschke O Jacques Joseph: father of modern facial plastic surgery Arch Facial Plast Surg 2008 10 05 300 303 18794406 10.1001/archfaci.10.5.300

[3] Gillies H D Plastic Surgery of the Face Based on Selected Cases of War Injuries of the Face Including Burns, with Original Illustrations London, UK Frowde 1920. Available at:https://archive.org/details/plasticsurgeryof00gilluoft. Accessed August 31, 2015

[4] Triana R J Jr Sir Harold Gillies Arch Facial Plast Surg 1999 1 02 142 143 10937096 10.1001/archfaci.1.2.142

[5] Spencer C R Sir Harold Delf Gillies, the otolaryngologist and father of modern facial plastic surgery: review of his rhinoplasty case notes J Laryngol Otol 2015 129 06 520 528 25858011 10.1017/S0022215115000754

[6] AAFPRS.Mission StatementAmerican Academy of Facial Plastic and Reconstructive Surgery (2015). Available at:http://www.aafprs.org/patient/about_us/pa_mission.html. Accessed August 31, 2015

[7] Larrabee W F Jr The International Federation of Facial Plastic Surgery Societies JAMA Facial Plast Surg 2013 15 06 403 404 24264259 10.1001/jamafacial.2013.2127

[8] Neaman K C Boettcher A K Do V H Cosmetic rhinoplasty: revision rates revisited Aesthet Surg J 2013 33 01 31 37 23277618 10.1177/1090820X12469221

[9] Ors S Ozkose M Ors S Comparison of various rhinoplasty techniques and long-term results Aesthetic Plast Surg 2015 39 04 465 473 25948068 10.1007/s00266-015-0497-5

[10] Gunter J P Landecker A Cochran C S Frequently used grafts in rhinoplasty: nomenclature and analysis Plast Reconstr Surg 2006 118 01 14e 29e 10.1097/01.prs.0000221222.15451.fc 16816668

[11] Parker Porter J Grafts in rhinoplasty: alloplastic vs. autogenous Arch Otolaryngol Head Neck Surg 2000 126 04 558 561 10772319 10.1001/archotol.126.4.558

[12] James S E Kelly M H Cartilage recycling in rhinoplasty: polydioxanone foil as an absorbable biomechanical scaffold Plast Reconstr Surg 2008 122 01 254 260 18594415 10.1097/PRS.0b013e31817741d3

[13] Greywoode J D Pribitkin E A Sonic rhinoplasty: histologic correlates and technical refinements using the ultrasonic bone aspirator Arch Facial Plast Surg 2011 13 05 316 321 21931085 10.1001/archfacial.2011.52

[14] Lopez M A Westine J G Toriumi D M The role of powered instrumentation in rhinoplasty and septoplasty J Long Term Eff Med Implants 2005 15 03 283 288 16022639 10.1615/jlongtermeffmedimplants.v15.i3.50

[15] Toriumi D M Dixon T K Assessment of rhinoplasty techniques by overlay of before-and-after 3D images Facial Plast Surg Clin North Am 2011 19 04 711 723, ix 22004862 10.1016/j.fsc.2011.07.011

[16] Cheng E T Perkins S W Rhytidectomy analysis: twenty years of experience Facial Plast Surg Clin North Am 2003 11 03 359 375 15062264 10.1016/S1064-7406(03)00023-3

[17] Miller T R Eisbach K J SMAS facelift techniques to minimize stigmata of surgery Facial Plast Surg Clin North Am 2005 13 03 421 431 16085288 10.1016/j.fsc.2005.04.007

[18] van der Lei B Cromheecke M Hofer S OP The purse-string reinforced SMASectomy short scar facelift Aesthet Surg J 2009 29 03 180 188 19608066 10.1016/j.asj.2008.10.010

[19] Atiyeh B S Dibo S A Costagliola M Hayek S N Barbed sutures “lunch time” lifting: evidence-based efficacy J Cosmet Dermatol 2010 9 02 132 141 20618559 10.1111/j.1473-2165.2010.00495.x

[20] Benedetto A V The lunchtime face lift Skinmed 2006 5 03 146 147 16687985 10.1111/j.1540-9740.2006.05607.x

[21] Sulamanidze M A Paikidze T G Sulamanidze G M Neigel J M Facial lifting with “APTOS” threads: featherlift Otolaryngol Clin North Am 2005 38 05 1109 1117 16214576 10.1016/j.otc.2005.05.005

[22] Rodriguez-Bruno K Papel I D Rhytidectomy: principles and practice emphasizing safety Facial Plast Surg 2011 27 01 98 111 21246461 10.1055/s-0030-1270427

[23] Castañares S Facial nerve paralyses coincident with, or subsequent to, rhytidectomy Plast Reconstr Surg 1974 54 06 637 643 4438462 10.1097/00006534-197412000-00001

[24] Prendiville S Weiser S Management of anesthesia and facility in facelift surgery Facial Plast Surg Clin North Am 2009 17 04 531 538, v 19900659 10.1016/j.fsc.2009.06.010

[25] American Society for Aesthetic Plastic Surgery.Cosmetic Surgery National Data Bank: 2006 statisticsAvailable at:http://www.surgery.org/sites/default/files/2014-Stats.pdf. Accessed August 20, 2015

[26] Lam S M Glasgold M J Glasgold R A Contemporary fat grafting Berlin, Heidelberg, Germany Springer 2008 217 224

[27] Naik M N Honavar S G Das S Desai S Dhepe N Blepharoplasty: an overview J Cutan Aesthet Surg 2009 2 01 6 11 20300364 10.4103/0974-2077.53092 PMC2840922

[28] Korchia D Braccini F Paris J Thomassin J Transconjunctival approach in lower eyelid blepharoplasty Can J Plast Surg 2003 11 03 166 170 24115863 10.1177/229255030301100311 PMC3792757

[29] de la Torre J I Martin S A De Cordier B C Al-Hakeem M S Collawn S S Vásconez L O Aesthetic eyelid ptosis correction: a review of technique and cases Plast Reconstr Surg 2003 112 02 655 660, discussion 661–662 12900629 10.1097/01.PRS.0000070985.40897.69

[30] Martin J J Jr Upper eyelid blepharoplasty with ptosis repair by levator aponeurectomy JAMA Facial Plast Surg 2015 17 03 224 225 25906318 10.1001/jamafacial.2015.0198

[31] Connell B F Lambros V S Neurohr G H The forehead lift: techniques to avoid complications and produce optimal results Aesthetic Plast Surg 1989 13 04 217 237 2596366 10.1007/BF01570355

[32] Yalçınkaya E Cingi C Söken H Ulusoy S Muluk N B Aesthetic analysis of the ideal eyebrow shape and position Eur Arch Otorhinolaryngol 2016 273 02 305 310 25348339 10.1007/s00405-014-3356-0

[33] Dayan S H Perkins S W Vartanian A J Wiesman I M The forehead lift: endoscopic versus coronal approaches Aesthetic Plast Surg 2001 25 01 35 39 11322395 10.1007/s002660010091

[34] Tower R N Dailey R A Endoscopic pretrichial brow lift: surgical indications, technique and outcomes Ophthal Plast Reconstr Surg 2004 20 04 268 273 10.1097/01.iop.0000131734.19609.15 15266139

[35] Bunagan M JK Banka N Shapiro J Hair transplantation update: procedural techniques, innovations, and applications Dermatol Clin 2013 31 01 141 153 23159183 10.1016/j.det.2012.08.012

[36] Dua A Dua K Follicular unit extraction hair transplant J Cutan Aesthet Surg 2010 3 02 76 81 21031064 10.4103/0974-2077.69015 PMC2956961

[37] Headington J T Transverse microscopic anatomy of the human scalp. A basis for a morphometric approach to disorders of the hair follicle Arch Dermatol 1984 120 04 449 456 6703750

[38] Rose P T Nusbaum B Robotic hair restoration Dermatol Clin 2014 32 01 97 107 24267426 10.1016/j.det.2013.09.008

[39] Avram M R Watkins S A Robotic follicular unit extraction in hair transplantation Dermatol Surg 2014 40 12 1319 1327 25418806 10.1097/DSS.0000000000000191

[40] Harris J A Follicular unit extraction Facial Plast Surg Clin North Am 2013 21 03 375 384 24017979 10.1016/j.fsc.2013.05.002

[41] Zakopoulou N Kontochristopoulos G Superficial chemical peels J Cosmet Dermatol 2006 5 03 246 253 17177748 10.1111/j.1473-2165.2006.00254.x

[42] Fulton J E Porumb S Chemical peels: their place within the range of resurfacing techniques Am J Clin Dermatol 2004 5 03 179 187 15186197 10.2165/00128071-200405030-00006

[43] Clark E Scerri L Superficial and medium-depth chemical peels Clin Dermatol 2008 26 02 209 218 18472062 10.1016/j.clindermatol.2007.09.015

[44] Fischer T C Perosino E Poli F Viera M S Dreno B; Cosmetic Dermatology European Expert Group.Chemical peels in aesthetic dermatology: an update 2009 J Eur Acad Dermatol Venereol 2010 24 03 281 292 19744174 10.1111/j.1468-3083.2009.03409.x

[45] Fulton J E Jr Dermabrasion, chemabrasion, and laserabrasion. Historical perspectives, modern dermabrasion techniques, and future trends Dermatol Surg 1996 22 07 619 628 8680784

[46] Alster T S Cutaneous resurfacing with CO2 and erbium: YAG lasers: preoperative, intraoperative, and postoperative considerations Plast Reconstr Surg 1999 103 02 619 632, discussion 633–634 9950554 10.1097/00006534-199902000-00040

[47] Goldberg D J Lasers for facial rejuvenation Am J Clin Dermatol 2003 4 04 225 234 12680801 10.2165/00128071-200304040-00002

[48] Trelles M A Mordon S Benítez V Levy J L Er:YAG laser resurfacing using combined ablation and coagulation modes Dermatol Surg 2001 27 08 727 734 11493296 10.1046/j.1524-4725.2001.01030.x

[49] Lipozenčić J Mokos Z B Will nonablative rejuvenation replace ablative lasers? Facts and controversies Clin Dermatol 2013 31 06 718 724 24160276 10.1016/j.clindermatol.2013.05.008

[50] Goldberg D J Non-ablative subsurface remodeling: clinical and histologic evaluation of a 1320-nm Nd:YAG laser J Cutan Laser Ther 1999 1 03 153 157 11360411 10.1080/14628839950516805

[51] Bitter P H Noninvasive rejuvenation of photodamaged skin using serial, full-face intense pulsed light treatments Dermatol Surg 2000 26 09 835 842, discussion 843 10971556 10.1046/j.1524-4725.2000.00085.x

[52] Jih M H Kimyai-Asadi A Fractional photothermolysis: a review and update Semin Cutan Med Surg 2008 27 01 63 71 18486026 10.1016/j.sder.2008.01.002

[53] Bodendorf M O Grunewald S Wetzig T Simon J C Paasch U Fractional laser skin therapy J Dtsch Dermatol Ges 2009 7 04 301 308 18761608 10.1111/j.1610-0387.2008.06845.x

[54] Paasch U Haedersdal M Laser systems for ablative fractional resurfacing Expert Rev Med Devices 2011 8 01 67 83 21158542 10.1586/erd.10.74

[55] Loesch M M Somani A-K Kingsley M M Travers J B Spandau D F Skin resurfacing procedures: new and emerging options Clin Cosmet Investig Dermatol 2014 7 231 241 10.2147/CCID.S50367 PMC4155739 25210469

[56] Donath A S Glasgold R A Glasgold M J Volume loss versus gravity: new concepts in facial aging Curr Opin Otolaryngol Head Neck Surg 2007 15 04 238 243 17620897 10.1097/MOO.0b013e32825b0751

[57] de Maio M The minimal approach: an innovation in facial cosmetic procedures Aesthetic Plast Surg 2004 28 05 295 300 15529203 10.1007/s00266-004-0037-1

[58] Ballin A C Brandt F S Cazzaniga A Dermal fillers: an update Am J Clin Dermatol 2015 16 04 271 283 26081021 10.1007/s40257-015-0135-7

[59] Jung D-W Kim Y H Kim T G Improvement of fat transplantation: fat graft with adipose-derived stem cells and oxygen-generating microspheres Ann Plast Surg 2015 75 04 463 470 26207545 10.1097/SAP.0000000000000580

[60] Locke M B de Chalain T MB Current practice in autologous fat transplantation: suggested clinical guidelines based on a review of recent literature Ann Plast Surg 2008 60 01 98 102 18281805 10.1097/SAP.0b013e318038f74c

[61] Goldfarb R M Shapiro A L Benefits of autologous fat grafting using fat mixed with platelet-rich fibrin matrix (PRFM) selphyl Am J Cosmet Surg 2012 29 01 62 64

[62] Yuksel E Choo J Wettergreen M Liebschner M Challenges in soft tissue engineering Semin Plast Surg 2005 19 03 261 270

[63] Saylan Z Facial fillers and their complications Aesthet Surg J 2003 23 03 221 224 19336082 10.1067/maj.2003.45

[64] Eppley B L Dadvand B Injectable soft-tissue fillers: clinical overview Plast Reconstr Surg 2006 118 04 98e 106e 10.1097/01.prs.0000232436.91409.30 16980841

[65] Levy R M Redbord K P Hanke C W Treatment of HIV lipoatrophy and lipoatrophy of aging with poly-L-lactic acid: a prospective 3-year follow-up study J Am Acad Dermatol 2008 59 06 923 933 19022099 10.1016/j.jaad.2008.07.027

[66] Bass L S Smith S Busso M McClaren M Calcium hydroxylapatite (Radiesse) for treatment of nasolabial folds: long-term safety and efficacy results Aesthet Surg J 2010 30 02 235 238 20442101 10.1177/1090820X10366549

[67] Carruthers J Fagien S Matarasso S L; Botox Consensus Group.Consensus recommendations on the use of botulinum toxin type a in facial aesthetics Plast Reconstr Surg 2004 114(6, Suppl):1S 22S 15507786 10.1097/01.PRS.0000144795.76040.D3

[68] Carruthers J A Lowe N J Menter M A A multicenter, double-blind, randomized, placebo-controlled study of the efficacy and safety of botulinum toxin type A in the treatment of glabellar lines J Am Acad Dermatol 2002 46 06 840 849 12063480 10.1067/mjd.2002.121356

[69] Binder W J Long-term effects of botulinum toxin type A (Botox) on facial lines: a comparison in identical twins Arch Facial Plast Surg 2006 8 06 426 431 17116793 10.1001/archfaci.8.6.426

[70] Small R Botulinum toxin injection for facial wrinkles Am Fam Physician 2014 90 03 168 175 25077722

[71] Trindade De Almeida A R Secco L C Carruthers A Handling botulinum toxins: an updated literature review Dermatol Surg 2011 37 11 1553 1565 21777338 10.1111/j.1524-4725.2011.02087.x

[72] Dessy L A Fallico N Mazzocchi M Scuderi N Botulinum toxin for glabellar lines: a review of the efficacy and safety of currently available products Am J Clin Dermatol 2011 12 06 377 388 21877763 10.2165/11592100-000000000-00000

[73] Rogers G M Allen R C Le Fort fractures New York, NY Springer 2012 283 295

[74] Zingg M Laedrach K Chen J Classification and treatment of zygomatic fractures: a review of 1,025 cases J Oral Maxillofac Surg 1992 50 08 778 790 1634968 10.1016/0278-2391(92)90266-3

[75] Strong E B Kim K K Diaz R C Endoscopic approach to orbital blowout fracture repair Otolaryngol Head Neck Surg 2004 131 05 683 695 15523449 10.1016/j.otohns.2004.05.017

[76] Dérand P Rännar L-E Hirsch J-M Imaging, virtual planning, design, and production of patient-specific implants and clinical validation in craniomaxillofacial surgery Craniomaxillofac Trauma Reconstr 2012 5 03 137 144 23997858 10.1055/s-0032-1313357 PMC3578652

[77] Suzuki T Kawamura H Kasahara T Nagasaka H Resorbable poly-L-lactide plates and screws for the treatment of mandibular condylar process fractures: a clinical and radiologic follow-up study J Oral Maxillofac Surg 2004 62 08 919 924 15278854 10.1016/j.joms.2004.01.016

[78] Eppley B L Zygomaticomaxillary fracture repair with resorbable plates and screws J Craniofac Surg 2000 11 04 377 385 11314387 10.1097/00001665-200011040-00019

[79] Eppley B L Sadove A M Havlik R J Resorbable plate fixation in pediatric craniofacial surgery Plast Reconstr Surg 1997 100 01 1 7, discussion 8–13 9207653 10.1097/00006534-199707000-00001

[80] Hammer B Schier P Prein J Osteosynthesis of condylar neck fractures: a review of 30 patients Br J Oral Maxillofac Surg 1997 35 04 288 291 9291270 10.1016/s0266-4356(97)90050-4

[81] Cabrini Gabrielli M A Real Gabrielli M F Marcantonio E Hochuli-Vieira E Fixation of mandibular fractures with 2.0-mm miniplates: review of 191 cases J Oral Maxillofac Surg 2003 61 04 430 436 12684959 10.1053/joms.2003.50083

[82] Tate J R Tollefson T T Advances in facial reanimation Curr Opin Otolaryngol Head Neck Surg 2006 14 04 242 248 16832180 10.1097/01.moo.0000233594.84175.a0

[83] Garcia R M Hadlock T A Klebuc M J Simpson R L Zenn M R Marcus J R Contemporary solutions for the treatment of facial nerve paralysis Plast Reconstr Surg 2015 135 06 1025e 1046e 10.1097/PRS.0000000000001273 26017609

[84] Tanzer R C Microtia—a long-term follow-up of 44 reconstructed auricles Plast Reconstr Surg 1978 61 02 161 166 622405 10.1097/00006534-197802000-00001

[85] Nagata S A new method of total reconstruction of the auricle for microtia Plast Reconstr Surg 1993 92 02 187 201 8337267 10.1097/00006534-199308000-00001

[86] Constantine K K Gilmore J Lee K Leach J Jr Comparison of microtia reconstruction outcomes using rib cartilage vs porous polyethylene implant JAMA Facial Plast Surg 2014 16 04 240 244 24763669 10.1001/jamafacial.2014.30

[87] Kelley P Hollier L Stal S Otoplasty: evaluation, technique, and review J Craniofac Surg 2003 14 05 643 653 14501322 10.1097/00001665-200309000-00008

[88] Stal S Klebuc K Advances in otoplasty: principles and techniques Key Issues Plast Cosmet Surg 2001 17 143 160

[89] Freeman S R Cohen J L New neurotoxins on the horizon Aesthet Surg J 2008 28 03 325 330 19083544 10.1016/j.asj.2008.03.006

[90] West T B Alster T S Effect of botulinum toxin type A on movement-associated rhytides following CO2 laser resurfacing Dermatol Surg 1999 25 04 259 261 10417577 10.1046/j.1524-4725.1999.08244.x

[91] Zimbler M S Holds J B Kokoska M S Effect of botulinum toxin pretreatment on laser resurfacing results: a prospective, randomized, blinded trial Arch Facial Plast Surg 2001 3 03 165 169 11497500 10.1001/archfaci.3.3.165

[92] Yamauchi P S Lask G Lowe N J Botulinum toxin type A gives adjunctive benefit to periorbital laser resurfacing J Cosmet Laser Ther 2004 6 03 145 148 15545098 10.1080/14764170410023767

[93] Carruthers J Carruthers A The effect of full-face broadband light treatments alone and in combination with bilateral crow's feet Botulinum toxin type A chemodenervation Dermatol Surg 2004 30 03 355 366, discussion 366 15008861 10.1111/j.1524-4725.2004.30101.x

[94] Coleman K R Carruthers J Combination therapy with BOTOX and fillers: the new rejuvnation paradigm Dermatol Ther 2006 19 03 177 188 16784517 10.1111/j.1529-8019.2006.00072.x

[95] Carruthers J Carruthers A A prospective, randomized, parallel group study analyzing the effect of BTX-A (Botox) and nonanimal sourced hyaluronic acid (NASHA, Restylane) in combination compared with NASHA (Restylane) alone in severe glabellar rhytides in adult female subjects: treatment of severe glabellar rhytides with a hyaluronic acid derivative compared with the derivative and BTX-A Dermatol Surg 2003 29 08 802 809 12859378 10.1046/j.1524-4725.2003.29212.x

[96] Carruthers J DA Glogau R G Blitzer A; Facial Aesthetics Consensus Group Faculty.Advances in facial rejuvenation: botulinum toxin type a, hyaluronic acid dermal fillers, and combination therapies—consensus recommendations Plast Reconstr Surg 2008 121(5, Suppl):5S 30S, quiz 31S–36S 18449026 10.1097/PRS.0b013e31816de8d0

[97] Garcia-Murray E Velasco Villasenor M L Acevedo B Safety and efficacy of RT002, an injectable botulinum toxin type A, for treating glabellar lines: results of a phase 1/2, open-label, sequential dose-escalation study Dermatol Surg 2015 41 01 S47 S55 25548845 10.1097/DSS.0000000000000276

[98] Flynn T C Advances in the use of botulinum neurotoxins in facial esthetics J Cosmet Dermatol 2012 11 01 42 50 22360334 10.1111/j.1473-2165.2011.00593.x

[99] Bly R A Chang S-H Cudejkova M Liu J J Moe K S Computer-guided orbital reconstruction to improve outcomes JAMA Facial Plast Surg 2013 15 02 113 120 23306963 10.1001/jamafacial.2013.316 PMC5951614

[100] Choi J W Kim N Clinical application of three-dimensional printing technology in craniofacial plastic surgery Arch Plast Surg 2015 42 03 267 277 26015880 10.5999/aps.2015.42.3.267 PMC4439584

[101] Azuma M Yanagawa T Ishibashi-Kanno N Mandibular reconstruction using plates prebent to fit rapid prototyping 3-dimensional printing models ameliorates contour deformity Head Face Med 2014 10 45 25338640 10.1186/1746-160X-10-45 PMC4213462

[102] Marro A Bandukwala T Mak W Three-dimensional printing and medical imaging: a review of the methods and applications Curr Probl Diagn Radiol 2016 45 01 2 9 26298798 10.1067/j.cpradiol.2015.07.009

[103] Chae M P Rozen W M McMenamin P G Findlay M W Spychal R T Hunter-Smith D J Emerging applications of bedside 3D printing in plastic surgery Front Surg 2015 2 25 26137465 10.3389/fsurg.2015.00025 PMC4468745

[104] Cheung K Voineskos S H Avram R Sommer D D A systematic review of the endoscopic management of orbital floor fractures JAMA Facial Plast Surg 2013 15 02 126 130 23370729 10.1001/jamafacial.2013.595

[105] Chen C T Lai J P Chen Y R Tung T C Chen Z C Rohrich R J Application of endoscope in zygomatic fracture repair Br J Plast Surg 2000 53 02 100 105 10878830 10.1054/bjps.1999.3289

[106] Czerwinski M Lee C The rationale and technique of endoscopic approach to the zygomatic arch in facial trauma Facial Plast Surg Clin North Am 2006 14 01 37 43 16466982 10.1016/j.fsc.2005.11.002

[107] Goodrich J T Sandler A L Tepper O A review of reconstructive materials for use in craniofacial surgery bone fixation materials, bone substitutes, and distractors Childs Nerv Syst 2012 28 09 1577 1588 22872276 10.1007/s00381-012-1776-y

[108] Bhumiratana S Vunjak-Novakovic G Concise review: personalized human bone grafts for reconstructing head and face Stem Cells Transl Med 2012 1 01 64 69 23197642 10.5966/sctm.2011-0020 PMC3513763

[109] Mendonça J J Juiz-Lopez P Regenerative facial reconstruction of terminal stage osteoradionecrosis and other advanced craniofacial diseases with adult cultured stem and progenitor cells Plast Reconstr Surg 2010 126 05 1699 1709 21042127 10.1097/PRS.0b013e3181f24164

[110] Marx R E Platelet-rich plasma: evidence to support its use J Oral Maxillofac Surg 2004 62 04 489 496 15085519 10.1016/j.joms.2003.12.003

[111] Cao Y Vacanti J P Paige K T Upton J Vacanti C A Transplantation of chondrocytes utilizing a polymer-cell construct to produce tissue-engineered cartilage in the shape of a human ear Plast Reconstr Surg 1997 100 02 297 302, discussion 303–304 9252594 10.1097/00006534-199708000-00001

[112] Friedman C D Future directions in biomaterial implants and tissue engineering Arch Facial Plast Surg 2001 3 02 136 137 11368669 10.1001/archfaci.3.2.136

[113] Langhals N B Urbanchek M G Ray A Brenner M J Update in facial nerve paralysis: tissue engineering and new technologies Curr Opin Otolaryngol Head Neck Surg 2014 22 04 291 299 24979369 10.1097/MOO.0000000000000062 PMC4171729

[114] Ledgerwood L G Tinling S Senders C Wong-Foy A Prahlad H Tollefson T T Artificial muscle for reanimation of the paralyzed face: durability and biocompatibility in a gerbil model Arch Facial Plast Surg 2012 14 06 413 418 22986911 10.1001/archfacial.2012.696

[115] Henstrom D K Masseteric nerve use in facial reanimation Curr Opin Otolaryngol Head Neck Surg 2014 22 04 284 290 25003843 10.1097/MOO.0000000000000070

[116] Fisher M D Zhang Y Erdmann D Marcus J Dissection of the masseter branch of the trigeminal nerve for facial reanimation Plast Reconstr Surg 2013 131 05 1065 1067 23629088 10.1097/PRS.0b013e318287a095

[117] Yoshioka N Tominaga S Masseteric nerve transfer for short-term facial paralysis following skull base surgery J Plast Reconstr Aesthet Surg 2015 68 06 764 770 25824195 10.1016/j.bjps.2015.02.031

[118] Cooper T M McMahon B Lex C Lenert J J Johnson P C Cross-facial nerve grafting for facial reanimation: effect on normal hemiface motion J Reconstr Microsurg 1996 12 02 99 103 8656408 10.1055/s-2007-1006461

[119] Biglioli F Facial reanimations: part I—recent paralyses Br J Oral Maxillofac Surg 2015 53 10 901 906 26188934 10.1016/j.bjoms.2015.06.023

[120] Frey M Michaelidou M Tzou C H Hold A Pona I Placheta E Proven and innovative operative techniques for reanimation of the paralyzed face Handchir Mikrochir Plast Chir 2010 42 02 81 89 20178074 10.1055/s-0029-1239590

[121] Hadlock T Cheney M L Facial reanimation: an invited review and commentary Arch Facial Plast Surg 2008 10 06 413 417 19018064 10.1001/archfaci.10.6.413

[122] Alam D S Chi J J Facial transplantation for massive traumatic injuries Otolaryngol Clin North Am 2013 46 05 883 901 24138744 10.1016/j.otc.2013.06.001

[123] Salibian A A Widgerow A D Abrouk M Evans G R Stem cells in plastic surgery: a review of current clinical and translational applications Arch Plast Surg 2013 40 06 666 675 24286038 10.5999/aps.2013.40.6.666 PMC3840172

[124] Matsumoto D Sato K Gonda K Cell-assisted lipotransfer: supportive use of human adipose-derived cells for soft tissue augmentation with lipoinjection Tissue Eng 2006 12 12 3375 3382 17518674 10.1089/ten.2006.12.3375

[125] Eun S-C Stem cell and research in plastic surgery J Korean Med Sci 2014 29 03 S167 S169 25473205 10.3346/jkms.2014.29.S3.S167 PMC4248001

[126] Francis M P Sachs P C Elmore L W Holt S E Isolating adipose-derived mesenchymal stem cells from lipoaspirate blood and saline fraction Organogenesis 2010 6 01 11 14 20592860 10.4161/org.6.1.10019 PMC2861738

[127] Balderson S Caputo J Colen B Girard L Hermann M Stem cell tourism: false hope for real moneyStem Cell Lines 2013;8(1). Available at:http://hsci.harvard.edu/stem-cell-tourism. Accessed September 22, 2015

[128] Hershcovitch M D Hom D B Update in wound healing in facial plastic surgery Arch Facial Plast Surg 2012 14 06 387 393 23165902 10.1001/2013.jamafacial.33

[129] Hom D B New developments in wound healing relevant to facial plastic surgery Arch Facial Plast Surg 2008 10 06 402 406 19018061 10.1001/archfaci.10.6.402

[130] Trindade A Djokovic D Gigante J Low-dosage inhibition of Dll4 signaling promotes wound healing by inducing functional neo-angiogenesis PLoS ONE 2012 7 01 e29863 22279550 10.1371/journal.pone.0029863 PMC3261161

[131] Carter M J Fylling C P Parnell L KS Use of platelet rich plasma gel on wound healing: a systematic review and meta-analysis Eplasty 2011 11 e38 22028946 PMC3174862

[132] Sclafani A P McCormick S A Induction of dermal collagenesis, angiogenesis, and adipogenesis in human skin by injection of platelet-rich fibrin matrix Arch Facial Plast Surg 2012 14 02 132 136 22006233 10.1001/archfacial.2011.784

[133] Qureshi A A Ross K M Ogawa R Orgill D P Shock wave therapy in wound healing Plast Reconstr Surg 2011 128 06 721e 727e 10.1097/PRS.0b013e318230c7d1 21841528

[134] Li Q Kao H Matros E Peng C Murphy G F Guo L Pulsed radiofrequency energy accelerates wound healing in diabetic mice Plast Reconstr Surg 2011 127 06 2255 2262 21311391 10.1097/PRS.0b013e3182131bb5

[135] Beanes S R Dang C Soo C Down-regulation of decorin, a transforming growth factor-beta modulator, is associated with scarless fetal wound healing J Pediatr Surg 2001 36 11 1666 1671 11685698 10.1053/jpsu.2001.27946

[136] Beanes S R Hu F Y Soo C Confocal microscopic analysis of scarless repair in the fetal rat: defining the transition Plast Reconstr Surg 2002 109 01 160 170 11786808 10.1097/00006534-200201000-00026

